# Regulatory Role of Serine59 in the Oligomeric Dynamics and Chaperone Function of αB-Crystallin

**DOI:** 10.21203/rs.3.rs-6787013/v1

**Published:** 2025-06-16

**Authors:** Tenzin Tender, Puttur Santhoshkumar, Leena Suleiman, Md Rejaul Hoq, K Krishna Sharma

**Affiliations:** 1 Department of Ophthalmology, University of Missouri, Columbia, MO – 65212 USA; 2 Electron Microscopy Core Facility, University of Missouri, Columbia – 65212 USA; 3 Department of Biochemistry, University of Missouri, Columbia, MO – 65212 USA

**Keywords:** αB-Crystallin, Oligomer, Chaperone, Phosphomimic, Interactions, Zeta potential

## Abstract

We previously demonstrated that deletion of the _54_FLRAPSW_61_ sequence, containing the key phosphorylation site Serine 59 (S59), resulted in a two-fold reduction in oligomeric mass and a ten-fold enhancement of αB-crystallin’s chaperone activity. This study examined whether targeted deletion (ΔS59) or phosphomimetic substitution (S59D) of S59 could replicate these effects. Using MALS analysis, we found that the average oligomeric mass decreased from 579 kDa in the wild type (αB-WT) to 556 kDa in ΔS59 and 434 kDa in S59D. Interestingly, the S59A variant had an increased mass of 611 kDa. All variants retained their chaperone function, but their efficiencies varied significantly. Specifically, S59D formed smaller, more polydisperse complexes that effectively suppressed aggregation when interacting with rapidly aggregating substrates. In contrast, ΔS59 and S59A created stable complexes with lysozyme, reducing precipitation and aggregate size. Zeta potential measurements indicated distinct surface charge profiles among the variants, but there was no clear correlation between these charges and their chaperone efficiency. Additionally, cytotoxicity assays conducted on ARPE-19 cells under oxidative stress showed that all S59 variants exhibited comparable protective effects against cell death relative to αB-WT. These results indicate that while S59 is not essential for oligomer formation or chaperone function, it plays an important role in modulating oligomer size and interactions with different substrates. Notably, the effects of S59D were measurable but did not replicate the enhanced functionality observed with the complete deletion of the 54–61 motif, reinforcing the significance of the N-terminal region.

## Introduction

The small heat shock protein αB-crystallin functions as a molecular chaperone, preventing the aggregation of misfolded proteins and maintaining cellular proteostasis.^1^ It plays a crucial role in the lens, muscle, and nervous system, and its dysregulation has been linked to cataracts, cardiomyopathies, and neurodegenerative diseases.^2^ A defining feature of αB-crystallin is its ability to form large, dynamic oligomeric assemblies, which are essential for its chaperone function.^3^ Understanding the molecular mechanisms governing αB-crystallin’s oligomerization and dynamic interactions is critical for elucidating its chaperone activity.

Human αB-crystallin consists of three key domains: the N-terminal region (NTR), the α-crystallin domain (ACD), and the C-terminal region (CTR). The ACD is the most conserved among small heat shock proteins (sHSPs), featuring a β-sandwich core crucial for dimerization.^2,4^ The NTR, a dynamic and hydrophobic region, regulates oligomer assembly and subunit interactions, while the CTR stabilizes oligomer formation and dictates its size.^5–7^ The N-terminal region serves as a critical interface for subunit interactions, as evidenced by studies showing that its complete deletion results in a tetrameric structure.^8^ Under environmental stress, oligomeric rearrangements expose hydrophobic regions, enhancing chaperone activity.^9^

Post-translational modifications, particularly phosphorylation, are pivotal in regulating αB-crystallin’s function.^3,10^ Three phosphorylation sites have been identified: Serine 19 (S19), Serine 45 (S45), and Serine 59 (S59).^10^ Among these, S59 is particularly significant as it resides within the critical _54_FLPRASW_61_ motif in the N-terminal region.^11^ Phosphorylation at this site has been shown to modulate oligomerization, solubility, and chaperone function.^10^ Previously, we demonstrated that deletion of this _54_FLPRASW_61_ region results in a two-fold reduction in oligomeric mass and a ten-fold increase in chaperone activity, underscoring its crucial role in regulating αB-crystallin’s structural and functional properties.^11^

Beyond its role as a chaperone, αB-crystallin exerts cytoprotective effects by inhibiting apoptosis.^12^ It protects cells from diverse stressors, including hydrogen peroxide, staurosporine, UV-A radiation, tumor necrosis factor, and etoposide.^13–15^ It interacts with key pro-apoptotic proteins such as Bcl, Bax, and p53, preventing their translocation to mitochondria and thereby inhibiting apoptosis.^14,16^ Given its essential role in maintaining cellular homeostasis, understanding the structural determinants that regulate αB-crystallin function is of significant interest.

**Fig. 1.**
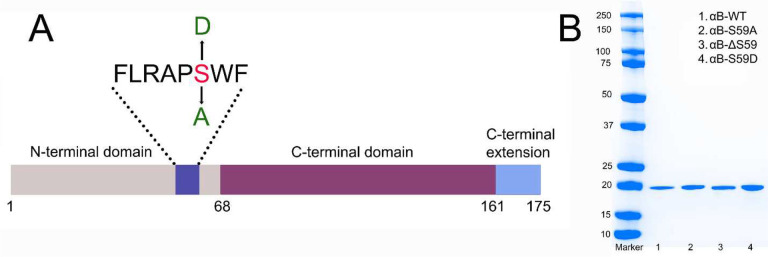
**A- A schematic diagram showing the αB-crystallin domains highlighting the previously deleted sequence (as reported in an earlier study).**^11^ The residue deleted/substituted in the current study is marked in red. B- SDS-PAGE of purified recombinant proteins used in the study and expressed in E.coli BL21(DE3)pLysS cells.

This study investigates the effects of S59 deletion and its phosphomimetic substitution (S59D) on αB-crystallin’s oligomerization and chaperone function. Building on our previous findings on the 54FLPRASW_61_ motif deletion, we sought to determine whether selectively removing S59 or introducing a phosphomimetic mutation (S59D) would elicit similar structural and functional effects. We generated three mutants—S59A, S59D, and ΔS59—through site-directed mutagenesis (Fig. 1A) in E. coli and assessed their impact on oligomeric assembly, chaperone function, and cell survival under stress conditions.

Our findings reveal that S59 does not directly dictate αB-crystallin’s oligomeric structure or chaperone function but plays a modulatory role. The chaperone activity of αB-crystallin and its mutants was substrate-dependent, and the effects of S59 deletion and phosphomimetic substitution were less pronounced than the αB-Δ54–61 mutant.^11^ These insights contribute to understanding αB-crystallin’s conformational dynamics and its implications in proteostasis and disease-associated dysregulation.

## Materials and Methods

### Overexpression and Purification of Wild-Type and Mutant αB-Crystallins

The expression and purification of wild-type (αB-WT) and mutant αB-crystallins (S59A, S59D, and ΔS59) were carried out following a previously published protocol,^17^ with modifications. Protein expression was induced using 0.5 mM IPTG in E. coli BL21(DE3)pLysS cells (Invitrogen, Carlsbad, CA, USA). After harvesting, the 1 L culture cell pellet was resuspended and lysed in 30 mL of lysis buffer (50 mM Tris-HCl, 2 mM EDTA, 0.1 M NaCl, pH 7.5) supplemented with 400 μL protease inhibitor cocktail III (Calbiochem-EMD Millipore, Billerica, MA, USA), 0.05 mL lysozyme (50 mg/mL) (Worthington, Lakewood, NJ, USA), and 5 units of benzonase nuclease (Sigma-Aldrich, St. Louis, MO, USA). The lysate was clarified by centrifugation at 18,000 × g for 30 minutes, followed by precipitation with 0.4 mL of 10% polyethyleneimine. A second centrifugation (18,000 × g, 30 minutes) was performed to remove nucleic acids and other contaminants. The supernatant was subjected to two rounds of purification using Q-Sepharose fast flow chromatography (Cytiva Sweden AB, Uppsala, Sweden), followed by size-exclusion chromatography on a HiLoad 16/60 Superdex column (GE Healthcare Biosciences, Piscataway, NJ, USA). The purified fractions were precipitated with 45% ammonium sulphate and further refined using a Superose 6 prep-grade column (Cytiva, Sweden). The final protein samples were pooled, concentrated, and stored at −80°C as 3 mg/mL aliquots. Protein concentration and molecular mass were determined using refractive index and multi-angle light scattering (MALS) detectors (Wyatt Technology, Santa Barbara, CA, USA). SDS-PAGE analysis confirmed sample purity exceeding 97% (Fig. 1B).

### Short-term anti-aggregation Assays

Alcohol dehydrogenase (ADH) (2.5 μM; Worthington, New Jersey, USA) and luciferase (0.5 μM; Promega, Madison, WI, USA), which rapidly aggregate and reach maximum scattering within 90 minutes at 37°C, were used as model substrates to assess the short-term chaperone activity of αB-WT and its mutants.^18^ Light scattering at 360 nm was measured over 90 minutes using a Spectramax i3 plate reader (Molecular Devices, San Jose, CA, USA) in the presence or absence of αB-WT or its mutants. Luciferase aggregation assays were conducted in PBS at 37°C with chaperone protein concentrations ranging from 0 to 40 μM. ADH aggregation was induced in a 0.25 mL assay buffer (PBS + 50 mM EDTA, pH 7.2) at 37°C, with chaperone protein concentrations ranging from 0 to 10 μM. The extent of substrate protein aggregation was calculated as a percentage relative to the control (aggregation in the absence of chaperone protein, set as 100%). The percentage aggregation at a given chaperone protein concentration was determined using the formula:

$$\text{Aggregation}\left(\%\right)=\frac{\text{Absorbance}\;\text{of}\;\text{sample}}{\text{Absorbance}\;\text{of}\;\text{control}}\times 100$$

To compare the relative chaperone efficiency of αB-WT and its mutants, EC_50_ values were determined, representing the chaperone concentration required to inhibit substrate aggregation by 50%. Non-linear regression analysis was performed using SigmaPlot v12.5 (Systat Software Inc., Palo Alto, CA, USA) with a four-parameter logistic curve function to obtain EC_50_ values from the aggregation plots.

### MALS analysis of ADH-Chaperone Protein Complexes

To assess the extent of complex formation, αB-crystallin and its mutants (100 μg) were individually incubated with ADH (75 μg) in 0.25 mL of PBS containing 50 mM EDTA at 37°C in a water bath for 60 minutes. Following incubation, the samples were centrifuged at 3,000 rpm for 10 minutes. A 100 μL aliquot of the supernatant, containing approximately 30 μg ADH and 40 μg chaperone protein, was injected into a TSK G5000PWXL column attached to a Shimadzu HPLC system equipped with UV and refractive index (RI) detectors and coupled to a MALS detector (DAWN EOS).^11^ The resulting peaks’ molar mass (Mw) and polydispersity were analyzed using ASTRA 6.1 software (Wyatt Technologies). A separate set of samples, processed identically but without incubation, was also analyzed for comparison.

### Long-term chaperone assay

Lysozyme (Worthington, New Jersey, USA) was used to evaluate the long-term chaperone activity of αB-WT and its mutants. The lysozyme aggregation assay (10 μM) was conducted in 1 mL of PBS containing 2 mM DTT (GoldBio, St. Louis, MO, USA) at 37°C, with chaperone protein concentrations ranging from 0 to 5 μM. The assay was monitored using a Shimadzu UV-Vis spectrophotometer. Under these conditions, lysozyme aggregation reaches full scattering within approximately 6 hours. The lysozyme-chaperone complexes’ effective radius and molecular mass were analyzed at 0 and 6 hours using a Nanobrook Omni system (Brookhaven Instruments, Holtsville, NY).

At the end of the chaperone assay, samples were centrifuged at 6,000 rpm for 20 minutes to separate the aggregates. The supernatant volume was adjusted to 1 mL, and an 80 μL aliquot was analyzed on a 4–20% SDS-PAGE gel (Genscript, Piscataway, NJ, USA) at 120 V for 90 minutes. Additionally, the precipitated fraction was resuspended to 0.25 mL, and 40 μL was loaded onto the gel to compare band intensities between the soluble and aggregated fractions.

### DLS analysis of lysozyme and chaperone protein incubation mixtures

Ten μM Lysozyme was treated with 10 μM αB-WT and its mutants (1:1) in 1 ml of PBS with 2 mM DTT at 37°C to assess the effective radius and molecular weight of the complex using Nanobrook Omni (Brookhaven Instruments, Holtsville, NY) at 0 and 6 hr. The effective radius and molecular weight of the individual proteins without incubation were also computed to gain an understanding of the alterations that the complex mixture exhibited.

### Transmission Electron Microscopy (TEM)

Transmission electron microscopy (TEM) was used to assess the morphology of the chaperone protein. Carbon-coated TEM grids were glow-discharged for 45 seconds at 15 mA to make the grid surface hydrophilic. For negative staining, 4 μL of the sample (0.05 mg/mL) was applied to the glow-discharged grid and incubated at room temperature for 1 minute. Excess liquid was blotted using filter paper, and 2% uranyl acetate (UA) was used for contrast enhancement. The UA was then washed off and blotted, and the grid was air-dried for 5 minutes before imaging. All negatively stained images were acquired using a JEM 1400 microscope operated at 120 kV, equipped with a Gatan Rio 9 CMOS camera.

### Zeta Potential Measurements

The zeta potential of αB-WT and its mutants was measured using the Nanobrook Omni (Brookhaven Instruments, Holtsville, NY) in PALS mode, equipped with a Uzgiris-type electrode assembly.^19^ Samples were prepared by dissolving 2 mg of protein in degassed sterile water to a final volume of 2 mL. Each measurement was conducted in five replicates, with 50 cycles per read, following the manufacturer’s recommendations.^20^

### Cell Viability Assay

Pre-authenticated ARPE-19 cells (obtained from ATCC, Manassas, VA, USA) were seeded into 96-well plates and cultured in Advanced-MEM/F12/DMEM-LG (2:1:1) media supplemented with Gibco^™^ Antibiotic-Antimycotic solution and 5% FBS. The cells were maintained under optimal conditions at 37°C with 5% CO_2_ until they reached 50–60% confluence. Once the desired confluence was achieved, the cells were treated with 6.5 mM sodium iodate (SI) in the presence or absence of αB-WT or its mutants to evaluate their ability to protect against SI-induced cytotoxicity.^16^ The plates were imaged at regular intervals using a Spectramax i3 plate reader (Molecular Devices, San Jose, CA, USA) to monitor the effect of SI on cell proliferation. The number of viable cells and the percentage of cell coverage area were quantified from brightfield images to assess cell density and morphological changes. Cell viability was further quantified using the CellTiter-Glo 2.0 assay (Promega, Madison, WI, USA), following the manufacturer’s protocol. The luminescence-based assay measures ATP levels, correlating with the number of metabolically active cells. The data presented represent the average of six wells, ensuring reproducibility and statistical significance. Additionally, cells from separate plates were stained using the ReadyProbes Cell Viability Imaging Kit (ThermoFisher) for fluorescence-based viability assessment. This kit allowed for a visual evaluation of live and dead cells to complement the quantitative data obtained from the luminescence assay.

## Results

### S59 does not directly influence oligomerization but modulates oligomer size through modification

**Fig. 2.**
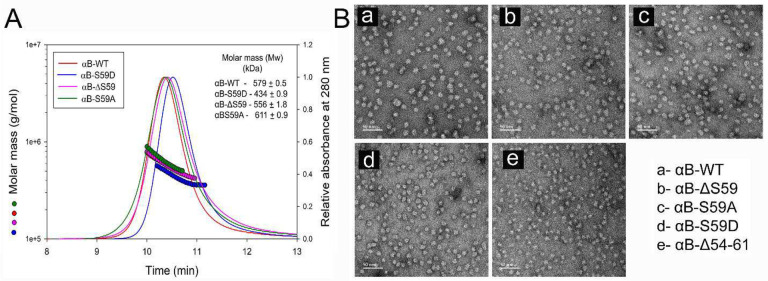
**A- Multi-angle light scattering (MALS) analysis of αB-WT and its mutants.** Proteins (0.15 mg in 0.05 mL) were incubated at 37°C for 1 hour prior to analysis. The molar mass distribution across the refractive index peaks and the polydispersity index (PDI) were determined from the MALS data using ASTRA (v6.1) software. Each analysis was performed three times using proteins prepared from independent batches, yielding consistent results. The data presented here is a representative example of one such analysis. B- Transmission electron micrographs of αB-WT and its mutants

In this study, we expressed αB-crystallin and its variants in E. coli BL21(DE3)pLysS cells and purified the proteins using gel filtration and ion-exchange chromatography. The introduced mutations did not affect protein solubility. The molecular mass (Mw) of αB-WT and its mutants was determined using HPLC coupled with multi-angle light scattering (MALS). The average oligomeric mass of αB-WT was 579 kDa (Fig. 2A). The deletion mutant (αB-ΔS59) exhibited a similar oligomeric mass (556 kDa), indicating that the absence of S59 does not significantly impact oligomerization. However, the phosphomimetic mutant (αB-S59D) displayed a 25% reduction in oligomeric mass (434 kDa) compared to αB-WT, suggesting that phosphorylation at this site regulates oligomer size by limiting subunit incorporation. In contrast, the S59A mutant (αB-S59A) exhibited an increased oligomeric mass (611 kDa), indicating that this substitution promotes subunit association. The TEM images (Fig. 2B) support these findings. The αB-S59D and αB-Δ54–61 show smaller oligomeric structures, with αB-Δ54–61 forming the smallest-sized oligomers. The oligomeric size of αB-ΔS59 was comparable to αB-WT, and that of αB-S59A appears slightly larger. Other oligomeric features were like αB-WT. These findings suggest that S59 is not directly involved in αB-crystallin oligomerization, but its modification (phosphorylation) plays a regulatory role in controlling oligomer size and subunit assembly.

### Phosphomimetic mutant (αB-S59D) enhances the chaperone-like activity of αB-crystallin

**Fig. 3.**
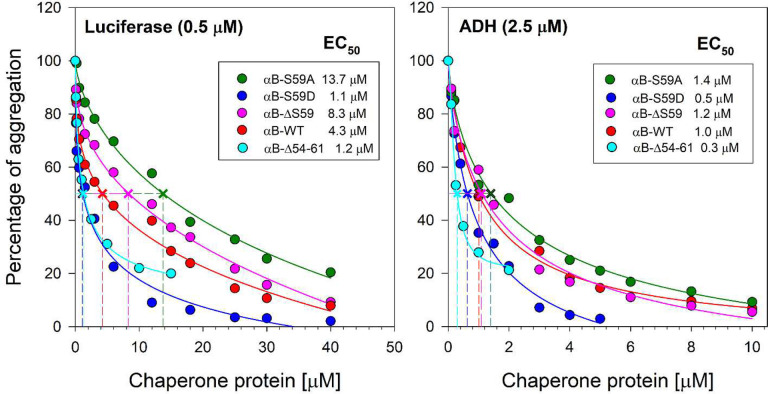
Comparison of the anti-aggregation activities of αB-WT and its mutants. The relative aggregation of luciferase and ADH was assessed in 0.25 mL assay buffer with varying concentrations of chaperone proteins. Aggregation was monitored by measuring light scattering at 360 nm using a plate reader, as described in the Methods section. Each experiment was repeated three times, with aggregation kinetics showing <3% variance. The EC_50_ values (chaperone concentration required to suppress substrate aggregation by 50%) shown in the figure were derived from a single representative experiment, based on absorbance readings at the 90-minute time point. Aggregation in the absence of chaperone protein was set as 100%, and all measured values at different chaperone concentrations are expressed relative to this baseline.

We compared the chaperone activities of αB-WT and its mutants (αB-ΔS59, αB-S59D, and αB-S59A) using luciferase and ADH as aggregation-prone substrates (Fig. 3). To quantify relative chaperone efficiency, EC_50_ values (Concentration of the chaperone protein required to suppress the aggregation of substrate protein by 50%) were estimated based on aggregation inhibition at the 90-minute assay time point. At 4.3 μM, αB-WT provided 50% protection against luciferase aggregation, while 1.0 μM was sufficient for the same level of protection against ADH aggregation. In comparison, αB-ΔS59 required 8.3 μM for luciferase and 1.2 μM for ADH, indicating reduced chaperone efficiency, particularly against luciferase aggregation. The phosphomimetic αB-S59D mutant exhibited a four-fold increase in chaperone activity with luciferase (EC_50_ = 1.1 μM) and a two-fold increase with ADH (EC_50_ = 0.5 μM) compared to αB-WT. Conversely, αB-S59A showed a significant loss in chaperone function, with EC_50_ values of 13.7 μM for luciferase and 1.4 μM for ADH, indicating reduced protective capacity. Additionally, chaperone assays with αB-Δ54–61 under identical conditions revealed an EC_50_ for luciferase (1.2 μM) comparable to αB-S59D, while for ADH, its chaperone efficiency was 1.6-fold higher than αB-S59D. These results indicate that S59 deletion (αB-ΔS59) does not significantly affect chaperone function with ADH but impairs protection against luciferase aggregation. In contrast, the phosphomimetic αB-S59D mutation enhances chaperone activity toward both substrates. While S59D mutation significantly improves chaperone function, its activity does not reach the levels of αB-Δ54–61 against ADH.

### MALS analysis of ADH and chaperone protein incubation mixtures

**Figure 4.**
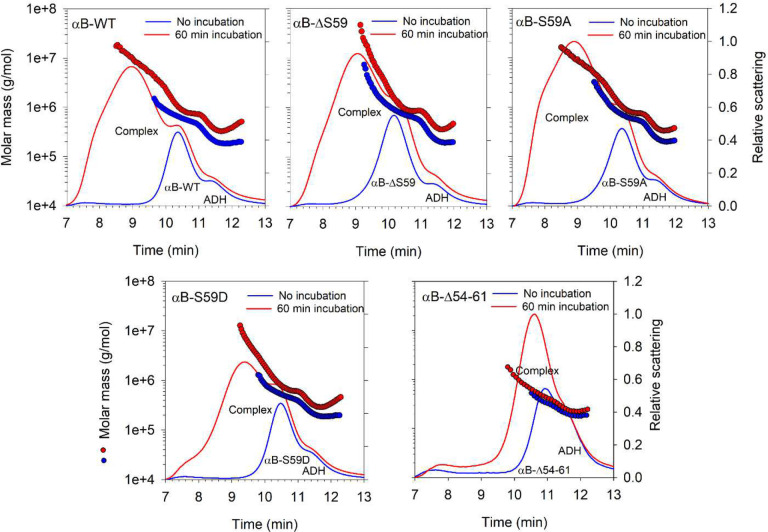
Multi-angle light scattering (MALS) analysis of ADH incubated with αB-crystallin variants. The figure shows molar mass distribution across the MALS elution profiles of ADH incubated with αB-WT and its mutants (αB-ΔS59, αB-S59D, αB-S59A, and αBΔ54–61) at 0 min and 60 min of incubation at 37°C. The elution peaks represent molecular mass (Mw) distribution, with earlier elution indicating larger protein complexes.

The multi-angle light scattering (MALS) analysis of ADH incubated with αB-WT and its mutants reveals distinct oligomerization patterns over time. The elution profiles show time-dependent shifts in molecular weight (Mw) and peak broadening, indicating dynamic chaperone-substrate interactions. At 0 min, distinct peaks corresponding to ADH and αB-crystallin variants are observed, with minor differences in elution times across mutants (Fig. 4). After 60 min of incubation at 37°C, the formation of larger protein complexes is evident, as seen by the emergence of broader, earlier eluting peaks, particularly in samples containing αB-WT, αB-ΔS59, and αB-S59A. The αB-S59D mutant, however, forms relatively smaller-sized complexes, as reflected by its less pronounced peak shift compared to αB-WT. The αBΔ54–61 mutant exhibits the smallest size complex formation, suggesting a distinct mechanism of chaperone function that may be prolonging substrate stabilization, reflected in its increased chaperone function. The average molar mass (Mw) and polydispersity (Mw/Mn) were estimated from light scattering detector signals using ASTRA 6.1 software for all identified protein peaks in Fig. 4, with results summarized in Table 1.

The multi-angle light scattering (MALS) analysis of αB-crystallin and its mutants in complex with ADH reveals a time-dependent increase in oligomeric mass and polydispersity, suggesting progressive binding and aggregation of unfolding substrate proteins. αB-WT + ADH exhibited a four-fold rise in average Mw (449.5 kDa to 1773.7 kDa) after 60 minutes, with a surge in polydispersity (1.37 to 2.61), indicating extensive oligomer reorganization and increased protein-binding capacity (Table 1). All other mutants also showed increased Mw and polydispersity, though to varying degrees. αB-ΔS59 + ADH showed a significant increase in Mw (668.5 kDa to 1569.4 kDa) with a rise in polydispersity (1.67 to 2.16), indicating a time-dependent complex formation as with αB-WT. αB-S59A + ADH exhibited the largest oligomeric mass after incubation (1794.2 kDa, PDI = 2.50), suggesting a greater tendency for aggregation. Phosphomimetic αB-S59D forms distinct, smaller-sized complexes: at 0 min, αB-S59D + ADH had a lower Mw (402.9 kDa) and polydispersity (1.25) compared to αB-WT. After 60 min, Mw increased to 1130.2 kDa with a polydispersity of 2.05, significantly lower than αB-WT and other S59 mutants. This suggests that αB-S59D undergoes a different oligomeric reorganization and binds unfolding ADH more efficiently, reducing the formation of large aggregates. αBΔ54–61 + ADH had the smallest Mw increase (270.1 kDa to 459.6 kDa) and lowest polydispersity (1.14 to 1.28), indicating the smallest-sized complexes among all variants, which may contribute to longer stability compared to larger complexes that precipitate earlier. This suggests that while Δ54–61 does not impair complex formation, it may enhance the stability of chaperone-substrate interactions over time. These findings indicate that S59 deletion (αB-ΔS59) does not drastically alter oligomerization, but its phosphomimetic substitution (αB-S59D) modulates complex formation, potentially delaying aggregation and precipitation.

### Long-Time Chaperone Assay with Lysozyme

**Fig. 5-:**
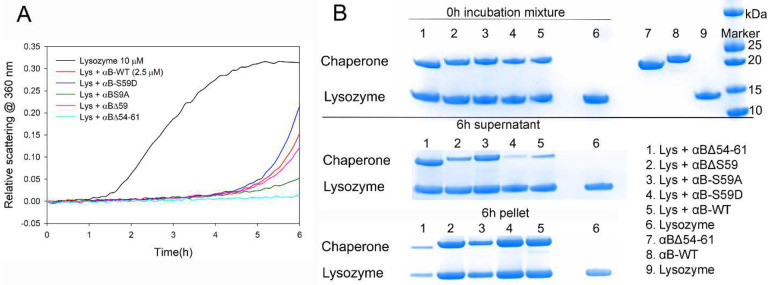
**A. Comparison of chaperone activity of αB-WT and its mutants using lysozyme**. This figure illustrates the chaperone activity assay of αB-WT and its mutants at a fixed concentration of 2.5 μM, using lysozyme (10 μM) as a long-term aggregation substrate. The lysozyme aggregation assay was conducted in 1 mL of PBS containing 2 mM DTT at 37°C, with chaperone concentrations ranging from 0 to 5 μM. The chaperone proteins exhibited concentration-dependent suppression of lysozyme aggregation. The aggregation inhibition pattern remained consistent across different concentrations, leading to the selection of 2.5 μM chaperone protein concentration for representation in the figure. B. SDS-PAGE Analysis of 6-hour lysozyme aggregation assay

The anti-aggregation activities of αB-WT and its mutants were evaluated under slow and progressive aggregation conditions using lysozyme (10 μM) as the substrate and 2 mM DTT as the aggregation-inducing agent in 1 mL PBS at 37°C for 6 hours. At a four-fold molar excess concentration, αB-WT exhibited moderate suppression of lysozyme aggregation, offering about 52% protection (Fig. 5A). Interestingly, the protection provided by S59 mutants against lysozyme differed markedly from their effects on ADH and luciferase substrates, suggesting a substrate-dependent modulation of chaperone activity. Among the mutants, αB-ΔS59 and αB-S59A demonstrated significantly higher efficiency than αB-WT, providing 60% and 84% protection, respectively, indicating that deletion or substitution at S59 promotes enhanced chaperone activity against lysozyme aggregation. In contrast, αB-S59D, which mimics phosphorylation at S59, was significantly less effective, offering only 30% suppression of lysozyme aggregation, implying that phosphomimetic modification at S59 weakens the chaperone’s ability to prevent lysozyme aggregation. Consistent with previous results using ADH and luciferase substrates, αB-Δ54–61 provided the highest anti-aggregation activity, offering >95% protection against lysozyme aggregation, reinforcing its role as a highly effective chaperone mutant.

Interestingly, the differences in protection observed with S59 mutants across different substrates suggest that modifications at S59 distinctly regulate the chaperone activity of αB-crystallin, influencing both oligomer stability and substrate binding dynamics. While αB-ΔS59 and αB-S59A promote efficient protection against lysozyme, αB-S59D reduces chaperone efficiency, likely due to altered oligomer-substrate interactions. These findings highlight the substrate-specific influence of S59 modifications on the chaperone function of αB-crystallin mutants and underscore the complexity of oligomeric dynamics in modulating chaperone efficiency.

Figure 5B displays the SDS-PAGE results of αB-WT and its mutants after the 6-hour lysozyme aggregation assay, comparing the supernatant and pellet fractions following centrifugation to assess chaperone participation and complex formation. αB-WT, S59D, and ΔS59 showed strong bands in the 6-hour pellet fraction (lanes 2, 4, and 5, respectively) and faint bands in the supernatant, indicating that these variants formed larger chaperone-substrate complexes that precipitated upon centrifugation, also suggesting increased lysozyme aggregation partitioning into pellet fraction compared to the control (lane 6). In contrast, S59A and Δ54–61 exhibited less intense bands forming from the pellet fraction (lanes 3 and 1, respectively) but strong bands in the supernatant, suggesting that these mutants formed smaller, more stable complexes that did not precipitate, indicating increased substrate stability and prevention of large aggregate formation. The lysozyme control (lane 6), where lysozyme was incubated without chaperone, showed a strong pellet band, reflecting extensive aggregation and precipitation without chaperone protection (compare to lane 1). Overall, these results indicate that αB-Δ54–61 has a greater capacity to interact with unfolding proteins in solution, and forms smaller and more stable complexes that resist precipitation, suggesting that the interaction of substrate proteins occurs at the oligomeric level rather than with individual chaperone subunits. These findings reinforce the idea that chaperone efficiency is modulated not only by structural changes at key residues (such as S59) but also by the ability of chaperone oligomers to engage with substrate proteins effectively, influencing aggregate stability and precipitation dynamics.

### DLS size analysis of lysozyme and chaperone protein incubation mixtures

To assess the extent of complex formation during chaperone assays, we measured the effective radius (nm) and molecular mass (g/mol) of the reaction mixtures prepared using equimolar amounts of substrate and chaperone protein. The assays were conducted under conditions specifically designed to prevent precipitate formation, which could otherwise interfere with accurate measurements. By maintaining an optimal substrate-to-chaperone ratio, we minimized the formation of visible aggregates that can lead to inaccurate data. This approach enabled a more reliable evaluation of complex formation dynamics and the efficiency of the chaperone in preventing substrate aggregation over time.

The cumulative and differential distribution of particles in the samples analyzed at 0 and 6 hours is shown in Fig. 1S. The mean effective radius (nm) and molecular mass (g/mol) were calculated from 8 independent scans using Brookhaven Particle Solutions V 3.5 software. The results from these scans are presented in Table 2, providing a quantitative comparison of particle size and mass changes over time, allowing for a comprehensive assessment of complex formation and chaperone efficiency. The data presented in Table 2 demonstrate that lysozyme alone showed a significant increase in effective radius from 0.2 ± 0.1 nm at 0 hours to 1264 ± 100 nm at 6 hours, accompanied by a massive increase in molecular mass from 0.3 × 10^6^ g/mol to 53300 × 10^6^ g/mol, indicating extensive aggregation over time. However, these values should be interpreted with caution due to the limited sensitivity of the instrument in measuring small molecules > 10 nm and potential interference from large aggregates scattering light at 6 hours.

In the presence of chaperone proteins, αB-WT moderately suppressed lysozyme aggregation, resulting in an increase in effective radius from 8.4 ± 0.3 nm to 134 ± 10 nm and an increase in molecular mass from 0.5 × 10^6^ g/mol to 294 × 10^6^ g/mol after 6 hours. Surprisingly, all mutants formed smaller complexes than αB-WT with lysozyme. Among the S59 mutants, αB-S59A exhibited the greatest anti-aggregation activity, with the radius increasing from 9.1 ± 0.1 nm at 0 hours to 42 ± 7 nm at 6 hours and the molecular mass increasing from 0.5 × 10^6^ g/mol to 22 × 10^6^ g/mol, suggesting that S59A forms smaller and more stable complexes that resist aggregation. αB-ΔS59 provided moderate suppression of aggregation, increasing the radius from 8.7 ± 0.1 nm to 60 ± 4 nm and the molecular mass increasing from 0.5 × 10^6^ g/mol to 45 × 10^6^ g/mol over 6 hours. In contrast, αB-S59D showed the least anti-aggregation activity, with the complex radius increasing from 9.0 ± 0.0 nm to 105 ± 6 nm and the molecular mass increasing from 0.5 × 10^6^ g/mol to 168 × 10^6^ g/mol after 6 hours. As expected, αB-Δ54–61 exhibited the most effective suppression of lysozyme aggregation, forming the smallest-sized and lowest-mass complexes over time, with the radius increasing from 10.4 ± 0.1 nm to 24 ± 1 nm and the molecular mass increasing from 0.7 × 10^6^ g/mol to 5 × 10^6^ g/mol after 6 hours, indicating superior chaperone activity. Interestingly, the higher oligomeric mass of the lysozyme and αB-Δ54–61 mixture at 0 hours (10.4 nm) compared to αB-Δ54–61 alone (7.3 nm) suggests a rapid interaction of the mutant with lysozyme, indicating a higher affinity of αB-Δ54–61 for lysozyme.

These findings suggest that modifications at S59 and deletions in the 54–61 region modulate the chaperone efficiency of αB-crystallin, influencing both complex size and aggregation suppression. The results further demonstrate that αB-Δ54–61 and S59A mutants provide better protection against aggregation, while S59D reduces chaperone efficiency, likely due to altered oligomer-substrate interactions.

### Zeta Potential Analysis and Its Relationship to Chaperone Function

The surface electrical charges on αB-WT and its mutants were evaluated using ζ potential measurements to assess differences in surface charge, which may influence protein-substrate interactions and complex formation. Our studies found that αB-Δ54–61 exhibited the highest average ζ potential, with a value of −21.51 ± 0.58 mV, indicating a higher negative surface charge compared to other variants. αB-WT had a ζ potential of −19.41 ± 0.78 mV, followed by αB-S59A with −14.78 ± 1.15 mV, αB-ΔS59 with −10.04 ± 1.02 mV, and αB-S59D, which exhibited the lowest ζ potential at −6.01 ± 0.24 mV. However, despite these differences in surface charge, no correlation was observed between the ζ potential values and the chaperone activity of αB-WT and its mutants. While αB-Δ54–61 exhibited the highest ζ potential and superior chaperone activity, αB-S59A, which had a lower ζ potential, also demonstrated improved anti-aggregation activity with lysozyme compared to WT. Conversely, αB-S59D, with the lowest ζ potential, showed reduced chaperone efficiency. These results suggest that net surface charge alone is not a determinant of chaperone efficiency and that other factors, such as oligomeric structure, substrate-binding dynamics, and conformational flexibility, play a more critical role in modulating the chaperone function of αB-crystallin and its mutants.

### αB-WT and S59 mutants suppressed the cytotoxicity action of NaIO_3_ on ARPE-19 cells

Sodium iodate (NaIO_3_) treatment induces oxidative stress that results in cytotoxicity in ARPE-19 cells^21^. This study investigated whether αB-WT and its S59 mutants could block the cytotoxic effects of NaIO_3_.^16^ In the initial phase of the assay, SI-treated wells (with or without chaperone proteins) showed no significant increase in cell number or coverage area at 16 hours. In contrast, the control wells (untreated) or those treated with chaperone proteins alone maintained a cell coverage of over 90%, indicating that αB-WT and its mutants were ineffective in suppressing the antiproliferative effects of SI during this period.

**Fig 6 -:**
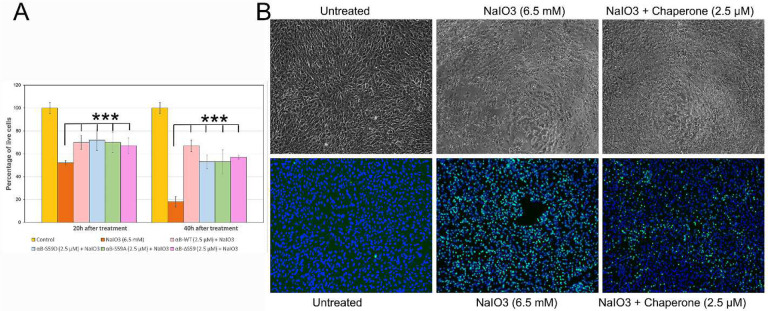
αB-crystallin and its S59 mutants protect ARPE-19 cells from NaIO_3_-induced cytotoxicity and morphological disruption. **A**- Bar graph showing the percentage of live cells at 20 and 40 hours after treatment with NaIO_3_ (6.5 mM), either alone or in combination with wild-type αB-crystallin (WT-αB) or its mutants S59D, S59A, and ΔS59 at 2.5 μM. NaIO_3_ significantly reduces cell viability, while co-treatment with αB-crystallin or its mutants improves survival, particularly at the 40-hour time point. Data are presented as mean ± SEM (n = 8) (* = p < 0.05). **B**- Brightfield (top) and fluorescence (bottom) images of ARPE-19 cells following NaIO_3_ and chaperone treatment. Brightfield images were captured 24 hours post-treatment, highlighting changes in cell morphology. Fluorescence images were acquired 36 hours post-treatment using the ReadyProbes Cell Viability Imaging Kit (ThermoFisher). Nuclei (blue) indicate total cell count, while dead cells (green) indicate cytotoxicity. Although overall viability improved with chaperone treatment, the number of dead cells was not significantly different among the chaperone-treated groups; thus, a representative image is shown.

The CellTiter-Glo 2.0 assay revealed no significant change in ATP levels (normalized to cell number or coverage area) at 16 hours (data not shown). However, by 20 hours, there was a 50% reduction in cell viability (ATP levels) in NaIO_3_-treated wells, while wells treated with SI and chaperone proteins showed only a 25% reduction in cell viability (Fig. 6A), suggesting partial protection offered by the chaperone proteins. At 40 hours, cell viability decreased by 80% in SI-treated wells, whereas in the presence of chaperone proteins, there was only a 50% reduction in cell viability compared to untreated cells. These results suggest that αB-WT and its mutants protect ARPE-19 cells from NaIO_3_-induced cytotoxicity over time. However, when tested at double the concentration (5 μM), there was no difference in the protection levels offered by the chaperone proteins (data not shown), suggesting that sufficient chaperone protein is already present at the initial concentration (2.5 μM) used to exert a maximum protective effect.

To determine whether the entry of chaperone proteins into the cells contributed to the observed protection, we performed Western blot analysis on the cell lysates to evaluate the band intensities of αB-WT and its mutants. The analysis revealed no significant difference in the band intensities across the different treatments, indicating that all chaperone variants entered the cells equally (data not shown). Furthermore, no significant difference in the cytoprotective activity was observed between αB-WT and its mutants in protecting ARPE-19 cells from NaIO_3_-induced cytotoxicity, suggesting that all variants provided comparable levels of protection in this model system.

The cell viability imaging studies corroborated these findings. The brightfield images of cells treated at 24 h showed that NaIO_3_ treatment significantly altered cellular morphology (Fig. 6B, upper panel centre), and the chaperone proteins protected cells from NaIO_3_-induced morphological changes (Fig. 6B, upper panel right). At 36 hours, in the absence of chaperone proteins, most of the cells were stained with green dye, indicating cell death (Fig. 6B). In contrast, in the presence of chaperone proteins, the number of dead cells was significantly reduced, suggesting that αB-WT and its mutants effectively protect ARPE-19 cells from NaIO_3_-induced cell death.

## Discussion

Alpha-crystallins, particularly αB-crystallin, are small heat shock proteins (sHSPs) that are vital in maintaining proteostasis and lens transparency.^5,22^ Their chaperone function, which involves binding and stabilizing partially unfolded proteins, is closely linked to their dynamic and polydisperse oligomeric structure.^9,23^ Previous studies have demonstrated that the N-terminal domain (NTD) of α-crystallin subunits plays a critical role in the formation of both homo- and hetero-oligomers.^3,24^ Modifications to specific regions or amino acid residues within this domain can significantly influence the protein’s oligomerization and structural organization.^3,25^ Our previous work has shown that deletion of residues 54–61 in αB-crystallin leads to a 39% reduction in oligomer size. Similarly, other investigators have reported a comparable decrease in size following deletion of residues 21–29.^26^ Furthermore, we have established that simultaneous deletion of residues 21–28 and 54–61 results in an approximately 80% reduction in oligomeric mass.^18^ These results, together with findings from other deletion studies, underscore the importance of specific NTD segments in mediating higher-order oligomeric assembly of αB-crystallin.^25,27^ These findings suggest that the NTD is critical not only for oligomerization but also for chaperone function and subunit interactions, even though there is limited evidence in the literature supporting a direct interaction of specific NTD residues with client proteins. Proteolytic digestion of αB-crystallin-lysozyme complex showed only a partial inhibition of chymotrypsin susceptible sites in the N-terminal domain of αB-crystallin.^28^ This could be due to the dynamic state of subunit interaction in the αB-crystallin oligomer that transiently provides access to protease-susceptible sites. At the same time, the client protein is bound elsewhere. Other investigators have reported that residues in the N-terminal domain of sHSP are involved in chaperone activity. Based on the loss of chaperone activity in mutants in the F24 to F27 region, it was reported that those residues are involved in chaperone activity.^29^ However, the enhanced chaperone activity shown by the 21–29 deletion mutant of αB-crystallin^26^ suggests that the loss of chaperone activity observed following mutation at the conserved F24–27 region might be due to structural and stability changes in the mutant protein. One can also argue that the 21–29 sequence in the N-terminal domain is likely a suppressor of chaperone activity, like that of the 54–61 region.

The present study focused on Serine 59 (S59), a major phosphorylation site located within the FLRAPSW sequence of αB-crystallin, which we had previously deleted^11^. The objective was to examine the specific contribution of S59 to αB-crystallin’s oligomerization and chaperone function. Our findings show that deletion of the S59 residue alone did not alter the oligomeric mass of αB-crystallin. However, the phosphomimetic substitution of S59 with aspartate (S59D) resulted in a 25% reduction in oligomeric mass (Fig. 2A). To our knowledge, this is the first study to report the effects of S59 deletion. These findings indicate that S59 is not directly involved in subunit interactions that dictate oligomer size but rather may be a regulator of oligomer size when it is post-translationally modified via phosphorylation. Consistent with our observations, previous studies have also reported reductions in oligomer size upon introducing single, double, or triple pseudo-phosphorylation mimics at Ser19, Ser45, and Ser59.^3,10,30,31^

Smaller oligomers and dimers of αB-crystallin are generally associated with enhanced chaperone activity, likely due to improved accessibility of substrate-binding regions.^5^ In our study, the chaperone activity of αB-crystallin mutants was highly substrate-dependent. The S59D mutant exhibited increased activity against ADH and luciferase, forming smaller, more efficient complexes that effectively suppressed aggregation (Fig. 3). However, this enhancement in chaperone activity was inconsistent across all substrates when tested with different mutants. αBS59D was the least effective against lysozyme, a slow-aggregating protein (Table 2). In contrast, the ΔS59 and S59A variants showed greater anti-aggregation activity against lysozyme, suggesting that S59 modifications influence substrate engagement in a substrate-specific manner, likely depending on the target protein’s aggregation kinetics and structural properties.^32^ These results highlight the complexity of substrate-chaperone interactions and support the idea that phosphorylation at S59 may confer conformational flexibility that selectively enhances interaction with rapidly aggregating substrates such as ADH and luciferase. Previous studies have also shown both decreased and increased chaperone activities of phosphomimetic mutants compared to αB-WT with respect to different targets tested, suggesting that the variations could be phosphorylation-induced modulation of chaperone activity.^10^ Another possibility for such differences could be the assay model and non-physiological temperatures used in some studies.^30,33,34^ We have carried out all chaperone assays at physiological temperatures. We found that the αB-S59A (control mutant) also showed both increased and decreased chaperone activity compared to αB-WT, suggesting a role for target protein aggregation kinetics and interaction in chaperoning efficiency. We noticed that proteins with higher chaperone activity show increased affinity towards substrates (recognizing early unfolding proteins) and form smaller-sized complexes (Table 2). Our DLS studies on the chaperone assay mixture with lysozyme support this. The oligomeric size of αB-Δ54–61 increased from 7.3 nm to 10.4 nm in the presence of Lysozyme at 0 min (when the reaction mixture was analyzed immediately after constituting), suggesting that αB-Δ54–61 had a strong affinity for lysozyme and probably recognized the early unfolding state of the substrate.

A related work on Hsp16.5 has shown that inserting a 14-residue sequence into the NTD increased both oligomer size and chaperone activity when tested with destabilized T4 lysozyme.^35,36^ In contrast, our results demonstrate that the smaller oligomers formed by the S59D mutant of αB-crystallin are associated with enhanced chaperone function when tested with ADH, luciferase, and lysozyme (Fig. 3 and Table 2). This observation contradicts previous reports in other sHSPs, where smaller oligomer size was linked to reduced chaperone activity when citrate synthase unfolding at 43 °C was used as substrate.^37^ The improved chaperone activity observed with S59D and ΔS59 is likely due to protein structural changes and subunit rearrangements that resulted in exposure of hydrophobic binding sites. Further studies are needed to determine those chaperone sites. While the 2- to 4-fold increase in anti-aggregation activity observed here is modest compared to the 100–5000-fold enhancement reported with immobilized αB-crystallin,^38^ it is still biologically meaningful.

Our findings also support the idea that NTD flexibility and oligomeric rearrangement affect chaperone efficiency. The Δ54–61 mutant consistently exhibited the strongest anti-aggregation performance across all tested substrates, forming small, stable complexes with minimal polydispersity (Figs. 3, 4, and 5). This reinforces the concept that targeted structural modifications within the NTD can be achieved by specific mutations, and the resulting gain of function may have applications where suppression of protein aggregation is warranted. Previously, we have shown that targeted mutation in the N-terminal domain results in functional Rescue of cataract-causing αA-G98R-crystallin.^39^

Studies with Spy chaperone have shown that selective introduction of negative charges enhances the chaperone activity.^35^ The increased chaperone activity shown by Spy was the result of the concerted action of hydrophobic sites and negative surface charges creating new binding pockets for the client protein. SHSPs have an overall negative charge under neutral conditions. The location of introduced negative charges on the sHSP can differentially affect its chaperone activity.^40^ The phosphomimetic S59D mutation introduces a negative charge, which we hypothesized would increase the net negative surface charge. The ζ potential is a feature of biomolecules that can be determined while suspended in liquid due to the arrangement of charged groups on the surface. For proteins, the ζ potential is determined by charged amino acids exposed to the solvent, which determines solution stability and prevents nonspecific aggregation among adjacent molecules due to charge repulsion. Surprisingly, ζ potential measurements revealed that S59D had the lowest negative surface charge among the tested variants. This could be due to the formation of the salt bridge between the S59D and a nearby Lys or Arg. It is yet to be confirmed which of the amino acids is involved in this interaction. Despite this, S59D demonstrated improved chaperone activity with luciferase and ADH. Conversely, Δ54–61, which exhibited the highest ζ potential, showed the greatest aggregation suppression. These results suggest that structural and conformational changes that result in active chaperone sites govern the chaperone function more than surface charge alone, as in the case of different mutants of Spy chaperone.^35^

In addition to their role in proteostasis, αB-crystallins are involved in suppressing apoptosis and oxidative stress. αB-crystallins can protect cells from oxidative damage and inhibit apoptosis by suppressing the processing of the pro-apoptotic protein caspase-3.^41^ It was found that the anti-oxidative activity was retained in αB-Δ54–61.^16^ Our experiments using ARPE-19 cells exposed to NaIO_3_ showed that αB-WT and all S59 mutants retained comparable anti-apoptotic activity (Fig. 6). While phosphorylation at S59 has been proposed as a regulatory switch for apoptotic signaling,^3,10^ our observation suggests that S59, in phosphorylated or unphosphorylated form, likely has no role in the pro- or anti-apoptotic activity or the anti-oxidative property of αB-crystallin, contrary to the observation made during earlier studies with brain astrocytes exposed to staurosporine.^42^ Previously, the overexpression of the S59E mutant, a phosphorylation mimic, was found to promote vinblastine-induced apoptosis in breast cancer cells, while the S59A mutant, which cannot be phosphorylated, displayed a protective effect.^43^ In another study, a pseudo phosphorylation mutant, S19E, S45E, and S59E, showed no anti-apoptotic activity.^44^ These results suggest that S59 is not essential for αB-crystallin’s cytoprotective effects under oxidative stress.

In summary, this study demonstrates that while S59 is not directly required for αB-crystallin oligomer formation, it plays a regulatory role in modulating oligomer size and enhancing chaperone activity through structural alterations after phosphorylation. The S59D phosphomimetic mutant improves chaperone function in a substrate-dependent manner without compromising or enhancing the cytoprotective capacity. Among all variants tested, the Δ54–61 mutant displayed the most robust anti-aggregation activity, underscoring the functional importance of the NTD. These findings deepen our understanding of how specific structural modifications influence αB-crystallin function and provide a foundation for developing therapeutic variants with enhanced chaperone efficacy.

## Supplementary Files

This is a list of supplementary files associated with this preprint. Click to download.

- PutturSupplement.pdf

## Figures and Tables

**Table 1.** MALS analysis of ADH and αB-WT and mutant incubation mixtures

	Sample	The average mass of all peaks Mw (kDa)	Polydispersity (Mw/Mn)
0-min incubation	αB-WT + ADH	449.5 (± 0.2%)	1.37 (± 0.4%)
	αB-S59D + ADH	402.9 (± 0.3%)	1.25 (± 0.4%)
	αB-ΔS59 + ADH	668.5 (± 0.2%)	1.67 (± 0.4%)
	αB-S59A + ADH	548.9 (± 0.5%)	1.47 (± 0.8%)
	αBΔ54-61 + ADH	270.1 (± 1.7%)	1.14 (± 2.3%)
60-min incubation	αB-WT + ADH	1773.7 (± 0.2%)	2.61 (± 0.5%)
	αB-S59D + ADH	1130.2 (± 0.2%)	2.05 (± 0.4%)
	αB-ΔS59 + ADH	1569.4 (± 0.4%)	2.16 (± 0.7%)
	αB-S59A + ADH	1794.2 (± 0.2%)	2.50 (± 0.6%)
	αBΔ54-61 + ADH	459.61 (± 1.3%)	1.28 (± 1.1%)

**Table 2.** Comparison of effective radius and molecular mass of lysozyme and its complexes with αB-crystallin variants at 0 and 6 hours

Samples	Effective radius (nm)		Molecular mass (g/mol)	
	0 hr	6 hrs	0 hr.	6 hrs
Lysozyme (1.8)	0.2 ± 0.1*	1264 ± 100*	0.3 ± 0 e^6^*	53300 ± 10000 e^6^*
Lysozyme + αB-WT (9.3)	8.4 ± 0.3	134 ± 10	0.5 ± 0 e^6^	294 ± 59 e^6^
Lysozyme + αB-S59D (8.3)	9.0 ± 0.0	105 ± 6	0.5 ± 0 e^6^	168 ± 24 e^6^
Lysozyme + αB-S59A (9.5)	9.1 ± 0.1	42 ±7	0.5 ± 0 e^6^	22 ± 7 e^6^
Lysozyme + αB-ΔS59 (9.2)	8.7 ± 0.1	60 ± 4	0.5 ± 0 e^6^	45 ± 7 e^6^
lysozyme + αB-Δ54-61 (7.3)	10.4 ± 0.1	24 ± 1	0.7 ± 0 e^6^	5 ± 0 e^6^

Note: Values marked with an asterisk (*) for lysozyme at 0 and 6 hours are unreliable due to instrument limitations in detecting small molecules and potential light scattering interference from large aggregates. The values in parentheses are the effective radius of the individual protein determined using the instrument, providing a reference for comparing the radius of the complexes formed during the incubation period.

## Data Availability

The data supporting this study’s findings are available from the corresponding authors upon reasonable request.
